# Antiarrhythmia drugs for cardiac arrest: a systemic review and meta-analysis

**DOI:** 10.1186/cc12852

**Published:** 2013-08-12

**Authors:** Yu Huang, Qing He, Min Yang, Lei Zhan

**Affiliations:** 1 The Third People’s Hospital of Chengdu, The Second Affiliated Hospital of Chengdu, Chongqing Medical University, Chengdu, China; 2 Department of Respiratory Disease, West China Hospital of Sichuan University, Chengdu, China; 3 Department of Intensive Care Unit, The Second Hospital of Anhui Medical University, Anhui, China; 4 Emergency Department of West China Hospital of Sichuan University, Chengdu 610041, China

**Keywords:** Heart arrest, Cardiopulmonary resuscitation, Antiarrhythmia agents, Outcome assessment

## Abstract

**Introduction:**

Antiarrhythmia agents have been used in the treatment of cardiac arrest, and we aimed to review the relevant clinical controlled trials to assess the effects of antiarrhythmics during cardiopulmonary resuscitation.

**Methods:**

We searched databases including Cochrane Central Register of Controlled Trials; MEDLINE, and EMBASE. Clinical controlled trials that addressed the effects of antiarrhythmics (including amiodarone, lidocaine, magnesium, and other new potassium-channel blockers) on the outcomes of cardiac arrest were included. Data were collected independently by two authors. The risk ratio of each outcome was collected, and meta-analysis was used for data synthesis if appropriate. Heterogeneity was assessed with the χ^2^ test and the *I*^*2*^ test.

**Results:**

Ten randomized controlled trials and seven observational trials were identified. Amiodarone (relative risk (RR), 0.82; 95% confidence interval (CI), 0.54 to 1.24), lidocaine (RR, 2.26; 95% CI, 0.93to 5.52), magnesium (RR, 0.82; 95% CI, 0.54 to 1.24) and nifekalant were not shown to improve the survival to hospital discharge compared with placebo, but amiodarone, lidocaine, and nifekalant were shown to be beneficial to initial resuscitation, assessed by the rate of return of spontaneous circulation and survival to hospital admission, with amiodarone being superior to lidocaine (RR, 1.28; 95% CI, 0.57 to 2.86) and nifekalant (RR, 0.50; 95% CI, 0.19 to 1.31). Bretylium and sotalol were not shown to be beneficial.

**Conclusions:**

Our review suggests that when administered during resuscitation, antiarrhythmia agents might not improve the survival to hospital discharge, but they might be beneficial to initial resuscitation. This is consistent with the AHA 2010 guidelines for resuscitation and cardiovascular emergency, but more studies with good methodologic quality and large numbers of patients are still needed to make further assessment.

## Introduction

Sudden cardiac arrest (SCA) is an emergency with high incidences but poor outcomes. Summary data indicate that the annual incidence of emergency medical service (EMS)-treated out-of-hospital cardiac arrest (OHCA) is about 50 to 55 per 100,000 persons in North America, and the annual incidence of in-hospital cardiac arrest (IHCA) ranges from three to six per 1,000 admissions. Fewer than 10% of them have been survived to discharge [1-4].

For the victims presenting with ventricular fibrillation (VF)/pulseless ventricular tachycardia (VT), antiarrhythmia agents are a kind of fundamental medication recommended by the resuscitation guidelines [5,6]. Some clinical trials studied the effects of antiarrhythmics on the outcomes of cardiac arrest. The number of participants in each study might be limited, and the individual studies might be different in methodologic quality, according to the study designs and methods. Several variables should be considered when evaluating the effects of resuscitation drugs, such as the scene of collapse or the quality of cardiopulmonary resuscitation (CPR). Although some antiarrhythmics have been recommended by the guidelines [5,6], evidence does not indicate that they could increase the survival from cardiac arrest, and more data are needed for the assessments of these drugs administered during CPR.

Besides the drugs recommended for routine use during CPR currently, such as amiodarone, lidocaine, and magnesium, we also supposed that the new potassium-channel blockers (such as ibutilide and so on), which emerged as important kinds of agents for various arrhythmias, might have the potential to be used during CPR [7-9]. Although reviews about antiarrhythmics used during CPR were undertaken as part of the ILCOR Consensus on CPR Science in 2010, however, new studies conducted after 2010 and also some new drugs were not included, and no meta-analyses were performed to evaluate the results quantitatively. Thus, we conducted the systematic review and meta-analysis to review the current literature, assess the relevant clinical trials quantitatively and qualitatively, and provide better evidence regarding the effects of antiarrhythmia agents on the outcomes of cardiac arrest.

## Methods

### Study eligibility

We indentified studies according to the following criteria: the studies were randomized controlled designs or prospective/retrospective cohort designs; the studies recruited adult (older than 18 years) cardiac arrest patients (OHCA and IHCA); all arrest rhythms were included; antiarrhythmic agents were administered during advanced cardiac life support (ACLS), including amiodarone, lidocaine, magnesium, other new potassium-channel blockers, such as nifecalant, ibutilide, dofetilide, and others, such as bretylium.

The studies had reported the following outcomes: ROSC; short-term survival: survival to hospital intensive care unit (ICU) admission for OHCA patients/survival to 24 hours for IHCA patients; survival to hospital discharge; and neurologic outcomes at discharge. Neurologic outcomes at hospital discharge were measured as cerebral performance category (CPC): good recovery (defined as a CPC score of 1 or 2) and unfavorable recovery (defined as a CPC score of 3, 4, or 5).

### Data sources

We searched the Cochrane Central Register of Controlled Trials (CENTRAL), MEDLINE (Ovid), and EMBASE. We also screened the reference lists of relevant trials and reviews. We searched the following databases for unpublished or ongoing studies: http://www.controlled-trials.com and http://clinicaltrials.gov.

We searched the combination of the keywords “heart arrest,” “sudden death,” “cardiopulmonary resuscitation,” “tachycardia, ventricular,” “ventricular fibrillation,” “arrhythmias, cardiac,” “advanced life support,” “antiarrhythmia agents,” “amiodarone,” “lidocaine,” “magnesium,” and “potassium-channel blockers.”

The searching was performed in October 2012, for all studies published in English between January 1948 and October 2012.

#### Study selection and data extraction

Two authors (Yu Huang and Qing He) independently screened all the titles and abstracts for eligibility. If we doubted whether a title or abstract should be included or excluded, then we read the full text to make a decision. The full texts were also read independently. Disagreements were solved by discussion with other authors, if any (Min Yang and Lei Zhan).

Two authors (Yu Huang and Qing He) extracted and collected data independently. The following data were abstracted: publication information mainly including first author’s last name and publication year; the settings of the study; the study design; characteristics of types of the included patients and the rhythms of arrest; information about sample collecting; the regimens of drug administration; and outcomes reported. Disagreements were resolved by discussion.

### Quality assessment of included studies

We assessed the methodologic quality of eligible trials by using the Risk of Bias tool recommended by the Cochrane Collaboration [10]. We assessed each trial across the following quality domains: (a) random-sequence generation; (b) allocation concealment; (c) blinding: according to special properties of CPR, we considered blinding adequate if the professional rescuers, the physicians in the hospital or the intensive care unit and the outcome assessors were blinded, regardless of the blinding of patients; (d) incomplete outcome data or loss to follow-up; and (e) selective reporting and any other potential threats to validity.

The assessment was performed by two authors (Yu Huang and Qing He) independently.

### Synthesis of results

The ROSC and survival outcomes were measured and derived as risk ratios (RRs) with 95% confidence intervals (CIs). Neurologic outcomes, grouped into the two categories of good recovery and unfavorable recovery, were also measured as RR with 95% CI to adapt them for meta-analysis. We synthesized the data by meta-analysis if available. Meta-analysis was performed by using Review Manager (RevMan 5.1). Heterogeneity was tested by using the χ^2^ test, and *P* ≤ 0.10 was considered significant. The *I*^*2*^ statistic also was used (*I*^*2*^% ≤ 25% for low, 25% <*I*^*2*^% < 50% for moderate, and *I*^*2*^% ≥ 50% for high). According to the variability of practice of CPR, including the different comorbidities of the included patients, the different treatment strategies and so on, we expected that a random-effects model would be suitable for this meta-analysis.

#### Subgroup analysis

We planned to perform subgroup analysis of OHCA and IHCA patients when applicable.

#### Sensitivity analysis

We planned to perform sensitivity analysis by excluding trials with high risk of bias when applicable.

## Results

**Figure 1:**
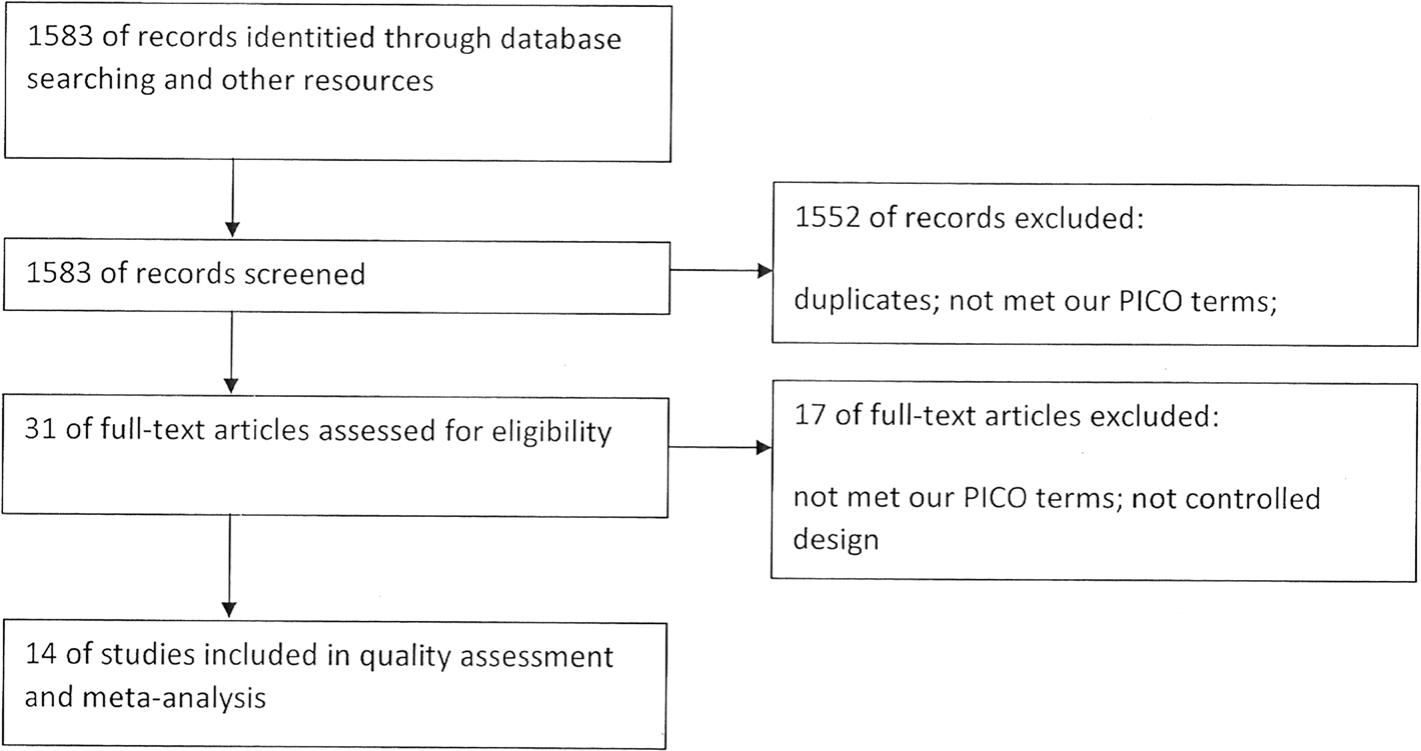
Flow diagram of literature search.

Our broad search identified 1,583 studies, and finally 14 were included after abstract screening and full-text reviewing (Figure 1) [11-24]. After analysis, we broadly categorized the studies into the following questions: antiarrhythmia agents (amiodarone/lidocaine/magnesium sulfate) versus placebo; amiodarone versus lidocaine; nifekalant versus lidocaine/amiodarone; and other drugs.

### Antiarrhythmia agents versus placebo

We identified eight eligible studies that compared the effects of antiarrhythmia agents versus placebo for cardiac arrest. Five of the included studies were randomized designed trials, and the other three were retrospective observational studies [11-18]. The details of study characteristics are shown in Table 1.

**Table 1:** Study characteristics

	**Study design**	**Setting and sample size**	**Inclusion of participants**	**Intervention**	**Control**	**Reported outcomes**
Kudenchuk *et al.*[11]	Randomized, double-blind, placebo-controlled study	Conducted in urban and suburban emergency medical service (EMS) systems in US, CPR was performed following the treatment protocols written in accordance with American Heart Association guidelines for advanced cardiac life support (ACLS) in 1982; 504 participants were enrolled	Adults with nontraumatic out-of-hospital cardiac arrest were eligible if ventricular fibrillation or pulseless ventricular tachycardia (on initial presentation or any time in the course of the resuscitation attempt) was present after three or more precordial shocks	300 mg of amiodarone diluted with dextrose, given intravenously	The diluent, polysorbate 80 as placebo	Survival to hospital intensive care unit admission; survival to hospital discharge
Skrifvars *et al*. [12]	Retrospective designed study	Conducted by Helsinki EMS systems; CPR was performed according to 2000 guidelines; 180 patients were enrolled	Adult OHCA patients with VF/pulseless VT resistant to three shocks	A bolus of 300 mg amiodarone after three ineffective shocks, an additional 150-mg dose might be administered	No amiodarone was administered	ROSC; survival to hospital admission; survival to hospital discharge
Fatovich *et al*. [15]	Double-blind, randomized controlled study	Undertaken at the ED of Royal Perth Hospital which served a population of 400,000 residents in urban setting; CPR was performed in accordance with the guidelines for clinical trials published by the Australian National Health and Medical Research Council; 67 patients were enrolled	All victims of OHCA receiving CPR, brought to the ED by the EMS system were eligible. Patients were excluded if they were already dead, not receiving CPR, already successfully resuscitated, or if the cardiac arrest was due to a noncardiac etiology	5 g MgSO_4_ (20 *M* in 10 ml), given as a bolus	10 ml 0.9% normal saline, given as a bolus	ROSC; 24-hour survival; survival to hospital discharge
Thel *et al*. [16]	Double-blind, randomized controlled study	Conducted by the Duke Hospital code team, CPR was performed according to the American Heart Association guidelines for ACLS; 152 participants were enrolled	All hospital inpatients in the intensive-care units and general wards who were at least 18 years old and treated for cardiac arrest by the Duke Hospital code team were eligible	2 g bolus of magnesium sulfate followed by an infusion of 8 g over 24-hour period	Matching placebo	ROSC; 24-hour survival; survival to hospital discharge; neurologic outcomes at hospital discharge (assessed by Glasgow Coma Scale score)
Allegra *et al*. [14]	Double-blind, randomized controlled study	Multicenter prehospital study clinical trial conducted in NJ, USA; standard ACLS algorithm was followed; 109 patients were enrolled	All patients with nontraumatic cardiac arrest who were 18 years of age or older and had VF refractory to three electroshocks	2 g magnesium sulfate	Equal volume of saline as placebo	ROSC; 24-hour survival; survival to hospital discharge
Hassan *et al.*[13]	Double-blind, randomized controlled study	Undertaken by the Leicestershire Ambulance and Paramedic Service which provided prehospital care to approximately 900,000 people in urban settings; CPR was performed according to ERC guidelines in 1992; 105 patients were enrolled	All adult patients (older than 18 years) with prehospital CA treated by EMS or in CA on arrival in the emergency department. The patient had either VF resistant to three shocks or a second episode of VF during a resuscitation cycle. CA patients related to trauma, hanging, or drowning were excluded	Magnesium sulfate (2 g or 8 m*M*) repeated with a further 2 g if the patient remained in VF after six shocks	Matched normal saline placebo	ROSC; 24-hour survival; survival to hospital discharge; Neurologic outcomes at hospital discharge (assessed by Glasgow Coma Scale score)
Harrision [17]	Retrospective design	Undertaken by EMS in urban and rural counties; 116 patients were enrolled	Adult patients with shock-resistant VF/VT	100-mg lidocaine bolus	No lidocaine given	Survival to admission; survival to discharge
Herlitz *et al.*[18]	Retrospective design	Conducted by two city hospitals in urban settings; 290 patients were enrolled	Adult cardiac-caused OHCA patients with VF/VT resistant to three shocks	50 mg lidocaine was given intravenously (could be repeated up to 200 mg)	No lidocaine given	ROSC; survival to coronary care unit admission; survival to discharge
Dorian *et al*. [19]	Randomized, double-blind, placebo-controlled study	The study was conducted under the auspices of, a multitiered out-of-hospital emergency-response system in Toronto; treatment protocols were in accordance with the American Heart Association guidelines for advanced cardiac life support; 347 participants were enrolled	Adult patients with nontraumatic out-of-hospital VF/other cardiac rhythms that converted to VF, VF was resistant to three shocks from an external defibrillator, at least one dose of intravenous epinephrine, and a fourth defibrillator shock	Amiodarone(5 mg/kg of estimated body weight diluted with dextrose), infused rapidly into a peripheral vein	Lidocaine (1.5 mg/kg at a concentration of 10 mg/ml), infused rapidly into a peripheral vein	Survival to hospital intensive care unit admission; survival to hospital discharge
Rea *et al.*[20]	Multicenter retrospective cohort study	Undertaken in three academic medical centers in the United States; CPR treatments and drug doses were according to 2000 AHA guidelines; 118 patients were enrolled	Patients experienced in-hospital cardiac arrest secondary to pulseless VT/VF were included. Pregnant women, prisoners, and patients younger than 18 years were excluded	Amiodarone administrated as recommended by the 2000 AHA guidelines	Lidocaine administered as recommended by the 2000 AHA guidelines	Survival to 24 hours; survival to hospital discharge
Amino *et al*. [21]	Retrospective observational study	Conducted by EMS system of Tokai University. The CPR protocol was adapted from ACLS algorithm recommended by AHA; 30 patients were enrolled	Adult out-of-hospital cardiac arrest patients with first defibrillation failure or VF recurrence were included	Nifekalant	Amiodarone	ROSC; survival to admission; survival to hospital discharge
Igarashi *et al*. [24]	Retrospective observational design	Conducted by Toho Omori University Hospital; 22 patients were enrolled	Adult out-of-hospital cardiac arrest patients with VF and unsuccessful defibrillation attempts by paramedics	Nifekalant(0.2-0.4 ml/kg)	Lidocaine(1–2 mg/kg)	ROSC; survival to discharge
Tahara et al. [23]	Retrospective, historic controlled design	Undertaken in urban settings in Yokohama, Japan; CPR treatments were according to 2000 AHA guidelines; 120 patients were enrolled	Patients who had out-of-hospital VF and were transferred to the university hospital, VF persisted after three shocks and a dose of epinephrine and another shock	Intravenous nifekalant (0.3 mg/kg)	Intravenous lidocaine (1.5 mg/kg)	Survival to admission; survival to hospital discharge
Shiga et al. [22]	Prospective observational study	Conducted in the cardiology departments of 10 hospitals in urban settings	Adult patients with VF when admitted to hospital	Nifekalant	Amiodarone	ROSC; short-term survival; survival to discharge
Nowak *et al*. [26]	Double-blinded, randomized	CPR treatments were consistent with American Heart Association protocols	OHCA patients	10 mg/kg of bretylium	Placebo	Survival to emergency department leaving
Olson *et al*. [25]	Randomized study	Conducted with the Milwaukee County Paramedic system	OHCA patients with refractory VF	5-10 mg/kg bretylium	1 mg/kg lidocaine	Survival to admission; survival to discharge
Kovoor *et al*. [27]	Randomized, double-blinded study	Conducted with the Ambulance Service of New South Wales	OHCA due to refractory VF	100 mg of sotalol	100 mg of lidocaine	Survival to admission; survival to discharge

One randomized controlled trial studied the use of amiodarone during CPR, and 504 patients were enrolled. Kudenchuk and colleagues performed a study conducted in urban and suburban emergency medical service (EMS) systems in the United States [11]; the CPR protocol was according to American Heart Association (AHA) guidelines published in 1982. OHCA patients (504) were enrolled, and 300 mg amiodarone or placebo was administered randomly if no resuscitation was gained after three shocks. The rescuers responsible for drug administration, advanced life support (ALS), or treatment in the emergency department (ED), and the investigators responsible for data collection and analyses, were all blinded adequately. The recipients of amiodarone were more likely to be resuscitated and survive to admission (RR, 1.27; 95% CI, 1.02 to 1.59); however, the proportion of patients who survived to discharge did not differ significantly in the two groups (RR, 1.02; 95% CI, 0.65 to 1.59). Thus although ROSC was not reported, amiodarone had significantly improved initial resuscitation compared with placebo, according to survival to hospital admission.

Another retrospective study conducted by the Helsinki EMS systems studied the use of undiluted amiodarone in the management of VF/pulseless VT resistant to three shocks [12]. No significant differences were shown in ROSC, survival to hospital admission, and discharge, but data demonstrated that the patients who received amiodarone had a more complicated prehospital course compared with those who did not receive amiodarone.

Four trials studied the effects of magnesium for cardiac arrest [13-16]; all were double-blinded randomized controlled trials. Three trials enrolled 370 OHCA patients [13-15]. In the case of Hassan *et al*. [13], the study was conducted in the county of Leicestershire, UK, and CPR was performed according to ERC guidelines published in 1992. Patients who had either VF resistant to three shocks or a second episode of VF during resuscitation were included. Magnesium sulfate (2 g) or placebo was given intravenously, and a repeated dose of 2 g magnesium sulfate might be given after six ineffective shocks. In the case of Allegra *et al*. [14], a multicenter prehospital study clinical trial conducted in New Jersey, USA. was found. Patients with VF refractory to three shocks were included, and magnesium sulfate (2 g) or placebo was given intravenously. In the case of Fatovich *et al*. [15], the study was undertaken at the ED of a large university hospital in Perth. OHCA patients were enrolled if they were still undergoing arrest when they arrived at the ED, and the patient management followed standard ALS guidelines (5 mg MgSO_4_ or 10 ml saline was given randomly at arrival at the ED). These three individual studies showed that no significant differences were found between magnesium and placebo groups in either ROSC or survival to discharge.

One trial was conducted by the Duke Hospital code team, and enrolled 67 IHCA patients from intensive care or general wards [16]. The primary diagnosis included circulatory/neurologic/respiratory disorders. The resuscitation protocol was according to AHA guidelines, MgSO_4_ (2-g bolus followed by an infusion of 8 g over a 24–hour period) or placebo given in a double-blinded manner. The results showed that in IHCA patients, no significant differences occurred between the magnesium and placebo groups in all outcomes.

Two retrospective studies compared lidocaine with placebo for OHCA patients with shock-resistant VF/VT [17,18]. In total, 406 patients were enrolled, and either individual study showed that lidocaine was superior to placebo in initial resuscitation but not survival to discharge.

We assessed the quality of the evidence by using the Cochrane Risk of Bias tool (see details in Table 2). In the five randomized trials, random sequence was generated by computer in the case of Allegra *et al*. [14], but no detailed information about the allocation concealment was found in the text. Sealed envelopes and central allocation were used for allocation in the case of Fatovich *et al*. [15], but no detailed information on randomized sequence generation was reported. No detailed information about random-sequence generation and allocation concealment could be found in the articles about other studies [11,13,16]. However, they are all randomized prospective designs, according to the texts, so unclear selection bias was considered [11,13-16]. Double-blinding was performed in all trials [11,13-16], and in the case of Kudenchuk *et al*. [11], the rescuers in the ED and the investigators for data collection and analyses were also blinded. Because considering the blinding of outcome assessors would not influence the analysis of ROSC or survival rate, a low risk of performing bias was considered. In the three retrospective studies, high risk of allocation and performing bias was considered [12,17,18]. Moreover, low risk of attribution and other bias was considered in all studies.

**Table 2:** Assessment methodologic quality

	**Random-sequence generation**	**Allocation concealment**	**Blinding**	**Incomplete outcome data addressed?**	**Free of selective reporting/other bias**	**Assessment of risk of bias across study**
Kudenchuk *et al*. [11]	Complete randomization was used according to the text, no details reported	Lack of details reported	Adequate	Yes	Yes	Low risk of bias
Skrifvars *et al*. [12]	High risk of allocation bias was considered according to the retrospective design		No blinding was performed	Yes	Yes	High risk of bias
Fatovich *et al*. [15]	Complete randomization was used according to the text, no details reported	Adequately performed	Adequate	Yes	Yes	Low risk of bias
Thel *et al*. [16]	Complete randomization was used according to the text, no details reported	Performed according to the text, lack of details reported	Adequate	Yes	Yes	Low risk of bias
Allegra *et al*. [14]	Random sequence generated by computer	Performed according to the text, lack of details reported	Adequate	Yes	Yes	Low risk of bias
Hassan *et al*. [13]	Complete randomization was used according to the text, no details reported	Sealed envelopes were used for allocation, adequate	Adequate	Yes	Yes	Low risk of bias
Harrision [17]	High risk of allocation bias was considered, according to the retrospective design		No blinding	Yes	Yes	High risk of bias
Herlitz *et al*. [18]	High risk of allocation bias was considered, according to the retrospective design		No blinding was performed	Yes	Yes	High risk of bias
Dorian *et al*. [19]	Complete randomization was used according to the text, no details reported	Adequately performed	Adequate	Yes	Yes	Low risk of bias
Rea *et al*. [20]	High risk of allocation bias was considered, according to the retrospective design		No blinding was performed	Yes	Yes	High risk of bias
Amino *et al*. [21]	Randomized controlled design, but lack of detailed information, unclear risk of allocation bias was considered		Blinding was performed, but lack of details	Yes	Yes	Unclear risk of bias
Igarashi *et al*. [24]	High risk of allocation bias was considered according to the retrospective design		No blinding was performed	Yes	Yes	High risk of bias
Tahara *et al*. [23]	High risk of allocation bias was considered according to the retrospective design		No blinding was performed	Yes	Yes	High risk of bias
Shiga *et al*. [22]	High risk of allocation bias was considered according to the prospective observational design		No blinding was performed	Yes	Yes	High risk of bias
Nowak *et al*. [26]	Randomization was performed, but lack of details was found	Lack of details reported	Double-blinding was performed	Yes	Yes	Unclear risk of bias
Olson *et al*. [25]	Randomization was performed, but lack of details was found	Lack of details reported	No blinding was performed	Yes	Yes	Unclear risk of bias
Kovoor *et al*. [27]	Quasi-randomization was considered, according to the text	Adequately performed according to the text, lack of details	Double-blinding was performed	Yes	Yes	Low risk of bias

**Figure 2:**
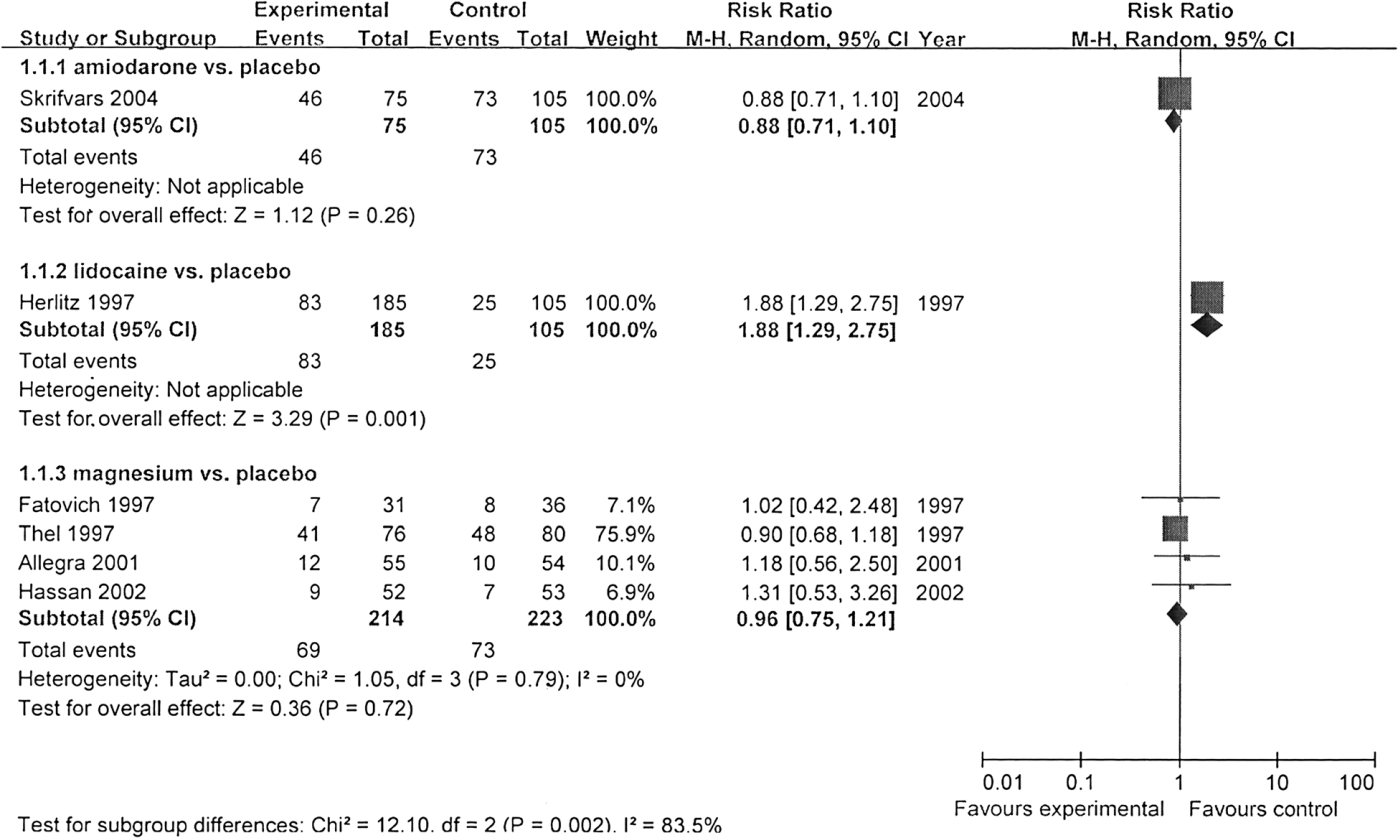
**Comparison of the effects of antiarrhythmics versus placebo.** Outcome: ROSC (return of spontaneous circulation). Subgroup analysis was performed according to different medications.

**Figure 3:**
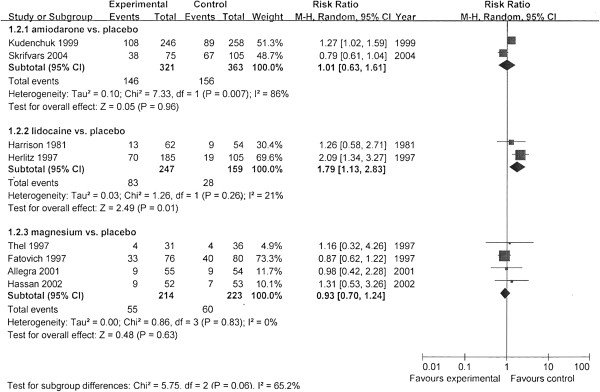
**Comparison of the effects of antiarrhythmics versus placebo.** Outcome: survival to hospital admission/24 hours. Subgroup analysis was performed according to different medications.

**Figure 4:**
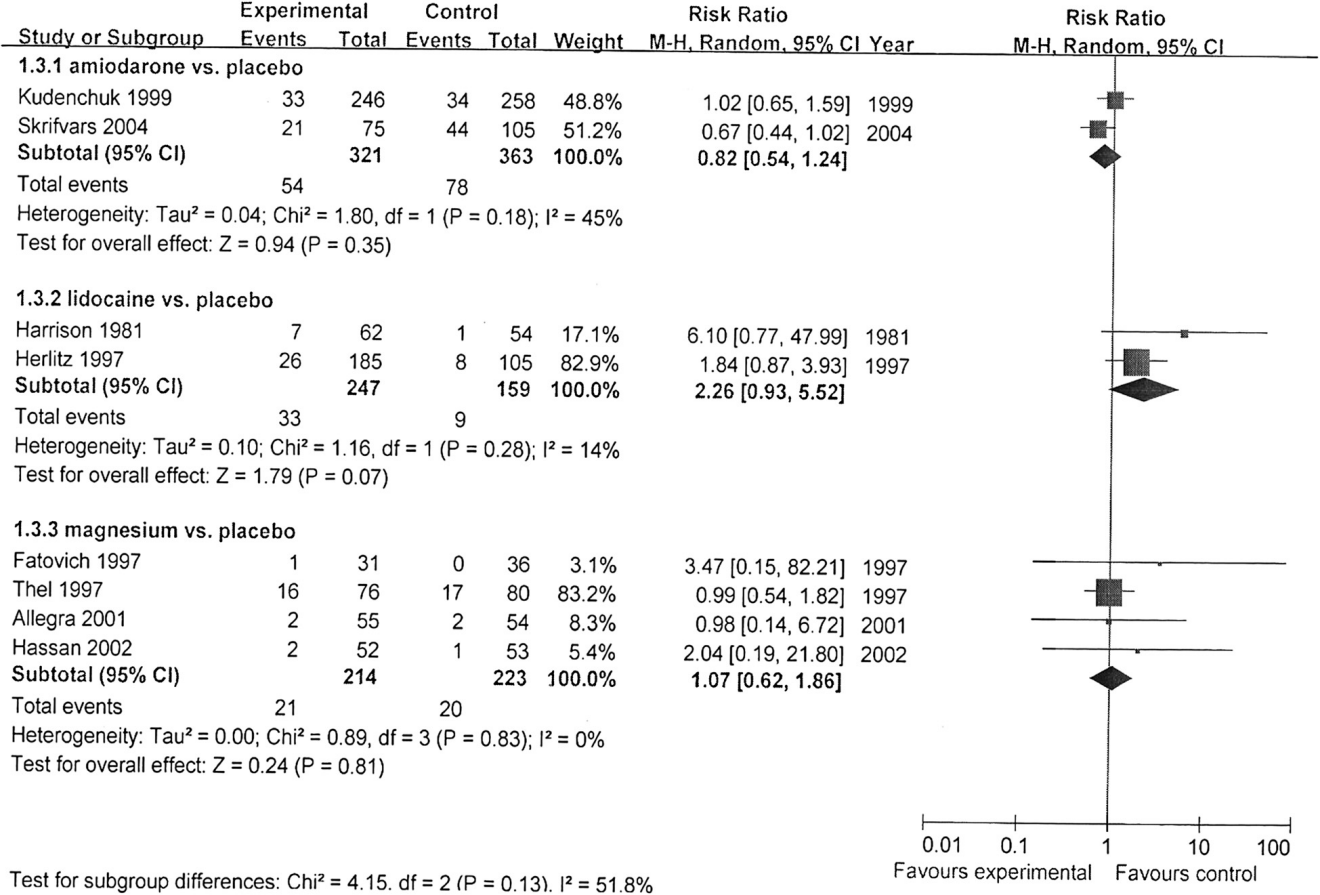
**Comparison of the effects of antiarrhythmics versus placebo.** Outcome: survival to hospital discharge. Subgroup analysis was performed according to different medications.

We performed meta-analysis by subgroups according to the different antiarrhythmics (Figures 2, 3, and 4). The rates of survival to hospital discharge were reported in all trials, whereas the other two outcomes were not. The pooled results showed that none of amiodarone (χ^2^ = 1.80; *P* = 0.18; *I*^*2*^ = 45%; RR, 0.82; 95% CI, 0.54 to 1.24) magnesium (χ^2^ = 0.89; *P* = 0.83; ^I2^ = 0; RR,1.07; 95% CI, 0.62 to 1.86) or lidocaine (χ^2^ = 1.16; *P* = 0.28; *I*^*2*^ = 14%; RR, 2.26; 95% CI, 0.93 to 5.52) had improved the survival to discharge. In the evaluation of outcomes of initial resuscitation, no difference was found in ROSC and survival to admission/24 hours between magnesium and placebo, but lidocaine was shown to improve initial resuscitation, according to the pooled results.

No trials reported data of neurologic outcomes assessed with CPC, so no meta-analysis was performed. In addition, only two trials reported data of neurologic outcomes measured by Glasgow Coma Scale score in OHCA and IHCA patients [13,16], and both trials showed that magnesium had not improved neurologic outcomes at hospital discharge compared with placebo.

### Amiodarone versus lidocaine

Two studies compared the effects of amiodarone and lidocaine during CPR (Table 1) [19,20]. Dorian and colleagues [19] performed a randomized study conducted under the auspices of the Toronto EMS system, and 247 patients were enrolled. OHCA patients with VF/pulseless VT refractory to defibrillation shocks were included, and the CPR was performed based on the 2000 AHA guidelines. Amiodarone (5 mg/kg) or lidocaine (1.5 mg/kg) was administered in a randomized, double-blinded manner. If VF persisted after a further shock, a second dose was given (1.5 mg/kg lidocaine; 2.5 mg/kg amiodarone), and resuscitation was continued. The rate of survival to admission was significantly higher in the amiodarone group compared with the lidocaine group (RR, 1.90; 95% CI, 1.16 to 3.11), however, no significant difference was found in the survival to discharge between the two groups (RR, 1.67; 95% CI, 0.57 to 4.88). Rea and colleagues [20] retrospectively reviewed the data of IHCA (pulseless VT/VF) from three large academic medical centers. Cardiac arrest was caused by circulatory or respiratory diseases. All CPR treatments and drug doses were according to 2000 AHA guidelines. The results showed that no difference was found in the proportion of patients alive 24 hours after arrest and at discharge between the two groups, the survival to ICU admission was reported. However, amiodarone was administered 8 minutes later on average, compared with lidocaine.

In Dorian *et al*. [19], adequate allocation concealment and binding was considered, according to the article, but no information about randomized-sequence generation was reported, so unclear risk of selection bias was considered. For the retrospective study [20], high risk of selection, performing, and detection bias was considered. Details are shown in Table 2.

**Figure 5:**
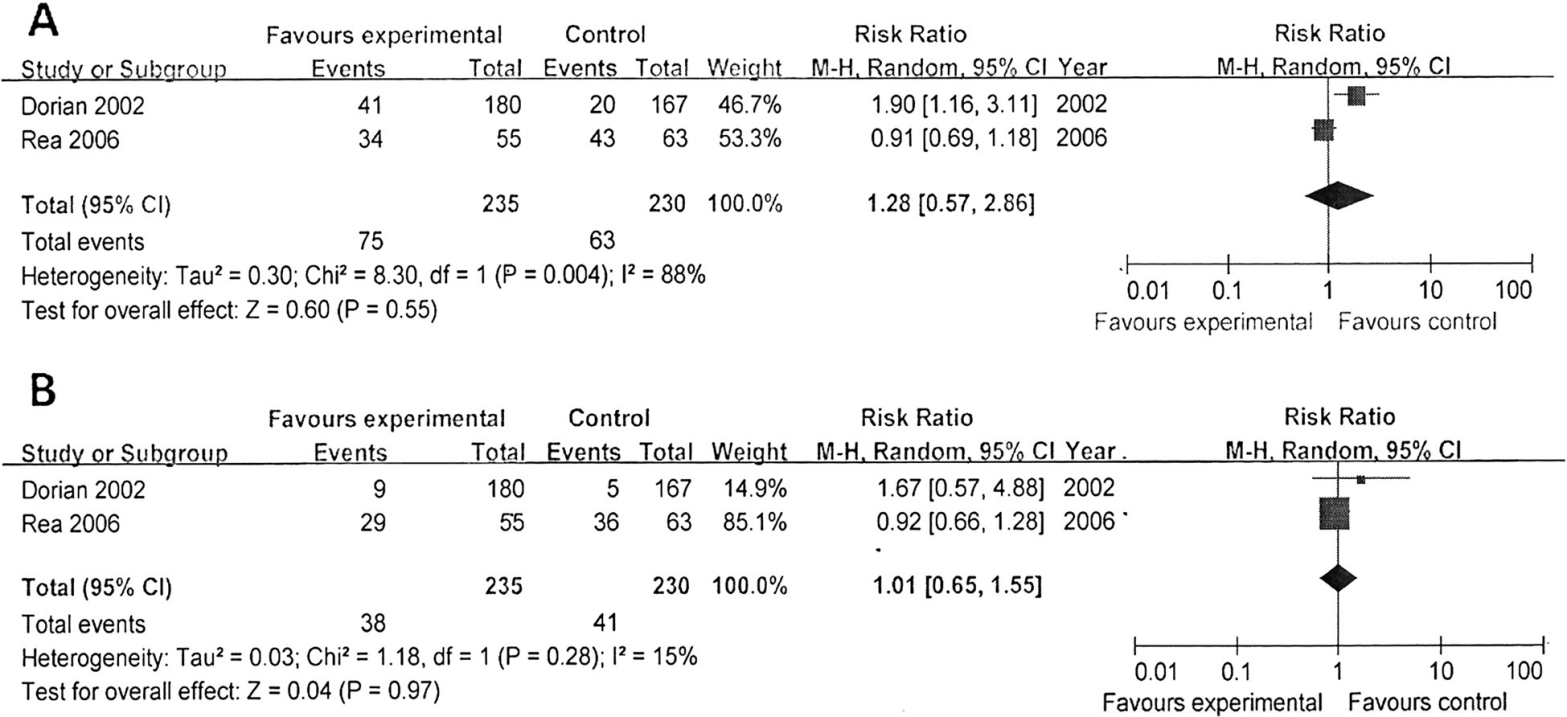
**Comparison of the effects of amiodarone versus lidocaine. (A)** Survival to hospital admission/24 hours. **(B)** Survival to hospital discharge.

Both of the two studies reported survival to discharge and survival to admission. The pooled results showed that no significant differences in survival to discharge (χ^2^ = 1.18; *P* = 0.28; I^2^ = 15%; RR, 1.01; 95% CI, 0.65 to 1.55) and also survival to admission (Figure 5).

### Nifekalant versus amiodarone/lidocaine

**Figure 6:**
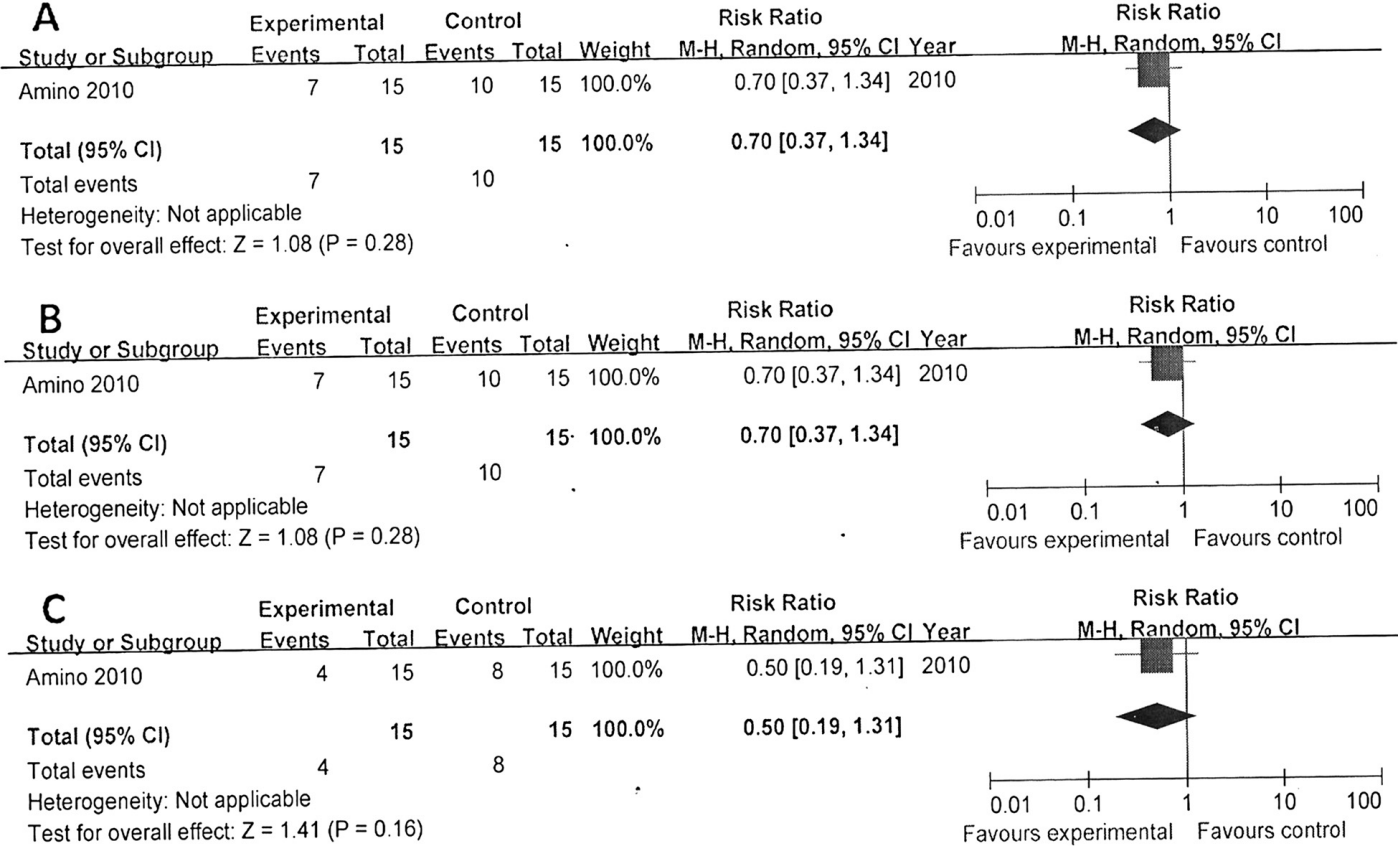
**Comparison of nifekalant with amiodarone. (A)** ROSC. **(B)** Survival to hospital admission/24 hours. **(C)** Survival to hospital discharge. ROSC, return of spontaneous circulation.

In our review, we attempted to study the use of new potassium-channel blockers during CPR, and only four trials that studied the effects of nifekalant were identified (Table 1) [21-24]. Amino and colleagues [21] compared nifekalant versus amiodarone for OHCA patients (shock-resistant VF) who were transferred to the EMS of Tokai University [21]. Thirty patients were enrolled, and the study drugs were given randomly; amiodarone was shown to be superior to nifekalant in all outcomes (Figure 6). Shiga and colleagues [22] conducted a prospective observational study to compare the effects of nifekalant versus lidocaine for IHCA (shock-resistant VF/VT) [22]. Tahara and colleagues [23] performed a retrospective, historical controlled study that enrolled OHCA patients with shock-resistant VF. Both studies showed that nifekalant significantly improved ROSC compared with lidocaine but not the live discharge. Additionally, Igarashi and colleagues [24] conducted a controlled study with only 22 patients to compare nifekalant and lidocaine for OHCA patients with VF. Nifekalant (0.2 to 0.4 ml/kg) or lidocaine (1 to 2 mg/kg) was given respectively after arrival at the ED and confirmation of VF. All patients died before discharge, but nifekalant was shown to improve the return of sinus rhythms compared with lidocaine.

**Figure 7:**
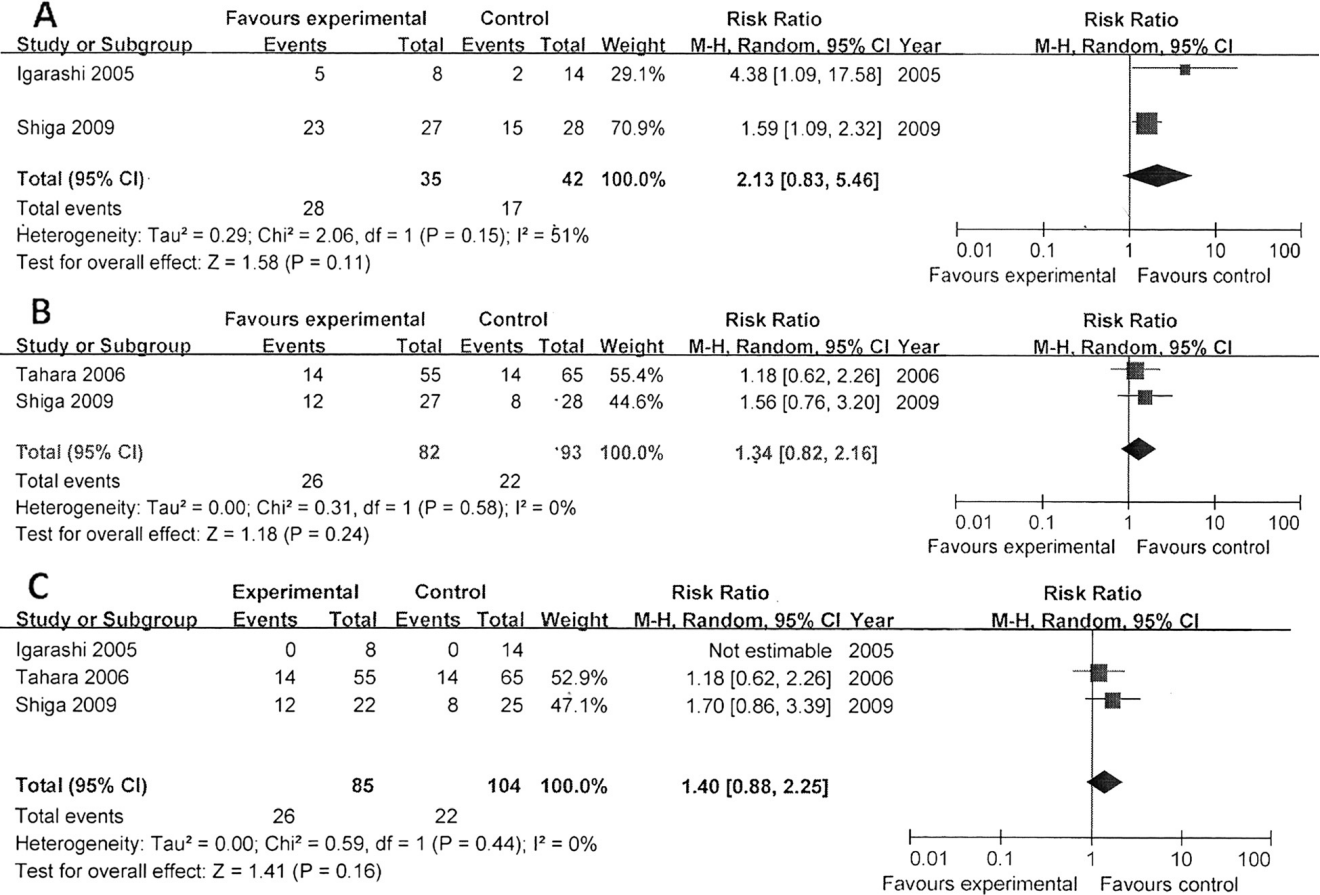
**Comparison of nifekalant versus lidocaine. (A)** ROSC. **(B)** Survival to hospital admission/24 hours. **(C)** Survival to hospital discharge. ROSC: return of spontaneous circulation.

In the assessments of methodologic qualities (Table 2), unclear risk of selection bias was considered for the randomized trial, but high risk of selection and performing bias was considered for the other two observational studies [21-23]. In the case of Igarashi *et al*., because no detailed information about study design was found in the text, a high risk of bias was considered for this study [24]. The overall pooled results showed that nifekalant significantly improved initial resuscitation compared with lidocaine (Figure 7), but no significant difference was found in the live discharge between the two groups (χ^2^ = 0.59; *P* = 0.44; *I*^*2*^ = 0; RR, 1.40; 95% CI, 0.88 to 2.25).

### Others

One randomized trial enrolled 91 out-of-hospital patients with VF refractory to four countershocks and compared the effect of bretylium with lidocaine [25]. Bretylium was not shown to have better effect than lidocaine on survival to hospital admission (RR, 1.01; 95% CI, 0.48 to 2.15) and survival to hospital discharge (RR, 0.45; 95% CI, 0.09 to 2.18). Another randomized double-blinded trial compared the effect of bretylium with placebo [26]. Bretylium was not shown to improve the proportion of patients who survived to leave the ED (RR, 4.28; 95% CI, 0.60 to 30.26).

In a randomized double-blinded trial of sotalol versus lignocaine in out-of-hospital VF patients [27], 129 patients with VF refractory to four shocks were enrolled, and the results showed that sotalol was not superior to lignocaine for improving survival to hospital admission (RR, 0.60; 95% CI, 0.33 to 1.10), survival to hospital discharge (RR, 0.33; 95% CI, 0.07 to 1.52), and neurologic outcome (RR, 0.13; 95% CI, 0.01 to 2.32).

#### Subgroup analysis

In the comparison of magnesium with placebo and amiodarone with lidocaine, both OHCA and patients were included, so we performed subgroup analysis of OHCA and IHCA patients.

In the comparison of magnesium with placebo, three double-blind, randomized controlled trials enrolled 281 OHCA patients, and magnesium was not shown to improve survival to hospital discharge (RR, 1.57; 95% CI, 0.41 to 6.05), ROSC (RR, 1.16; 95% CI, 0.71 to 1.89), and survival to ICU admission (RR, 0.92; 95% CI, 0.68 to 1.24) [13-15]. One double-blind, randomized controlled trial enrolled 67 IHCA patients, and the results showed that magnesium had not improve survival to hospital discharge (RR, 0.99; 95% CI, 0.54 to 1.82), ROSC (RR, 0.90; 95% CI, 0.68 to 1.18), or survival to 24 hours (RR, 1.16; 95% CI, 0.32 to 4.26) [16].

In the comparison of amiodarone with lidocaine, one double-blind, randomized controlled trial with 347 patients showed that amiodarone improved the survival to ICU admission (RR, 1.90; 95% CI, 1.16 to 3.11) in OHCA patients compared with lidocaine [19]. However, in IHCA patients, the other retrospective study that enrolled 118 patients, showed no significant difference in survival to 24 hours between the two treatments (RR, 0.91; 95% CI, 0.69 to 1.18) [20]. No significant difference was found between the effects of amiodarone and lidocaine on survival to hospital discharge (RR, 0.99; 95% CI, 0.54 to 1.82) in both OHCA and IHCA patients [19,20].

#### Sensitivity analysis

In the comparisons of amiodarone with placebo and amiodarone with lidocaine, only one trial could be identified if the other trials with high risk of bias were excluded [11,19]. In the comparison of amiodarone with placebo, amiodarone was shown to improve ROSC rate and survival to admission (RR, 1.27; 95% CI, 1.02 to 1.59) compared with placebo; however, the survival to discharge did not differ significantly in the two groups (RR, 1.02; 95% CI, 0.65 to 1.59) [11]. These results were consistent with those synthesized before exclusion of the retrospective designed study, which was considered to be with high risk of bias.

In the comparison of amiodarone with lidocaine, when the retrospective designed study with high risk of bias was excluded, only one randomized blinded trial was included, and no significant difference was found in the survival to discharge between the two groups (RR, 1.67; 95% CI, 0.57 to 4.88), but the survival to admission was significantly better in the amiodarone group compared with the lidocaine group (RR, 1.90; 95% CI, 1.16 to 3.11) [19]. This was not consistent with the totally pooled results. This result might come from the design of two studies. In the retrospective designed study, no randomization and blinding were performed, so high risk of selection bias and detection bias were considered. However, statistical heterogeneity (*I*^*2*^ = 88%) and clinical heterogeneity (the retrospective designed study enrolled IHCA patients) were found.

## Discussion

In our review, we included all adult nontraumatic patients. The included studies were all conducted in urban or suburban settings, and the medical treatment was performed by European/American/Asian EMS systems or EDs, respectively. The CPR treatment during ALS was according to AHA or ERC guidelines published in 2000 or before. The majority of participants’ rhythms were presented as VF/VT.

In the comparison of magnesium versus placebo, four studies with good methodologic quality were included [13-16], and no significant differences could be found in ROSC, survival to admission, and survival to hospital discharge between the two treatments. In subgroups of OHCA and IHCA patients, no significant differences were found in all outcomes. Although two trials included few patients with other rhythms, we did not perform subgroup analysis for these rhythms because of such small sample size [15,16]. Thus, our results demonstrate that magnesium has no significant benefits when used for CPR, for either OHCA or IHCA.

In the assessments of the effects of amiodarone, no improvement of survival to admission was found in ROSC, survival to admission, and survival to hospital discharge when treated with amiodarone according to the pooled results. But when excluding a retrospective observational study, one randomized trial that had enrolled 504 patients and was considered to have good quality showed that amiodarone had improved the survival to admission but not the survival to discharge, compared with placebo [11]. All high statistical (*I*^*2*^ = 86%), methodologic, and clinical heterogeneity was concerned between the two studies included in the synthesis. The heterogeneity derived from the retrospective study with high risk of bias [12], and in this study, the prehospital condition was more severe in the amiodarone group [20]. So the pooled results of survival to admission must be interpreted with caution. According to the high-quality study [11], amiodarone is still likely to be beneficial to initial resuscitation, although the effect size is rather small (RR, 1.27; 95% CI, 1.02 to 1.59).

We also identified two studies that had compared amiodarone with lidocaine. The pooled results showed that no significant differences could be found in the survival to hospital discharge and survival to admission/24 hours between the two study groups. However, one good-quality study indicated that amiodarone was superior to lidocaine in initial resuscitation (evaluated by the survival to admission) [19]. This was inconsistent with the results reported from the other retrospective designed study and also the pooled result [20]. According to sensitivity analysis, the pooled results did not seem to be robust. However, the retrospective observational study included IHCA patients, whereas the RCT included OHCA patients, and it was considered to be with high risk of bias. Moreover, in the observational study, amiodarone was administered later than lidocaine, and it was not administered with the recommended doses to all the patients. So it is suggested that the reduced demonstrated effects of amiodarone on survival to 24 hours in this study is due to such clinical heterogeneity. Significant statistical heterogeneity (*I*^*2*^ = 88%) was also considered between these two studies. Thus, in the comparison of amiodarone with lidocaine, the pooled result of the survival to admission still must be interpreted with caution. We generally suggest the use of amiodarone instead of lidocaine, according to the findings of the randomized trial.

Two studies with high risk of bias compared lidocaine with placebo [17,18], and showed that no significant differences were found in those alive at discharge between the two groups. Although it is indicated that lidocaine has the potential to improve initial resuscitation compared with placebo, the quality of evidence is low, and we suggest that the replacement of lidocaine with amiodarone in clinical use seems to be appropriate.

Moreover, we suggest that the new potassium-channel blockers might have the potential to be used during CPR. However, after our literature search, no more trials were identified, except for four trials that focused on the effects of nifekalant, and they were all conducted in Japan [21-24]. The pooled results of three low-quality trials indicated that nifekalant was superior to lidocaine in initial resuscitation but not in the survival outcomes [22-24]. By contrast, a randomized trial with small sample size showed that nifekalant was inferior to amiodarone in either initial resuscitation or survival outcomes [21]. Thus, no present evidence can support the use of such potassium-channel blocker instead of amiodarone. Although nifekalant may be superior to lidocaine in initial resuscitation, considering the low quality of evidence, it is still hard to suggest the use of nifekalant.

Some studies focused on the use of bretylium and sotalol, but the very limited data showed no benefits of the two drugs.

Above all, all antiarrhythmic drugs were not shown to be significantly beneficial for cardiac arrest. However, because many variables might affect the practice of resuscitation, such as the cause of arrest, the response interval of the EMS system, the chest-compression fraction, the quality of CPR, and postresuscitation treatment. Thus, regarding these issues, the retrospective design of a study might bring high risk of overall bias through the study, including allocation, performing, and detection bias. All of these might have the potential to affect the demonstration of treatment effect. Therefore, according to the high-quality studies [11,19], it is suggested that amiodarone still seems to be superior to placebo (RR, 1.27; 95% CI, 1.02 to 1.59) and lidocaine (RR, 1.90; 95% CI, 1.16 to 3.11) for improving initial resuscitation assessed by the survival to admission, although the effect sizes are not big.

For the lack of data, we only performed subgroup analysis according to OHCA and IHCA in the comparison of magnesium with placebo and amiodarone with lidocaine. Similar results were found in both subgroups in the comparison of magnesium with placebo for all outcomes. Although in the comparison of amiodarone with lidocaine, the results were not consistent in the two subgroups in the analysis of initial resuscitation, this inconsistence might derive from the nonrandomization of the observational study.

Limitations in our review include that little evidence can be found after searching the literature. In the subgroups of amiodarone versus placebo, amiodarone versus lidocaine, and nifekalant versus amiodarone, only one or two controlled trials could be found. In total, 433 patients were included in the pooled results of the subgroup of magnesium versus placebo. Therefore, the results derived from present literature were rather underpowered to appraise certain ideas according to the sample sizes.

The second limitation is related to the methodologic quality of individual evidence. Many of the included studies were observational cohort studies, and especially, retrospective design. We suggest that adequate randomization and blinding of the rescuers is important to reduce the allocation, performing, and detection bias in clinical practice of CPR; thus, high risk of bias might be considered for these individual observational studies.

The third limitation is related to the heterogeneity, especially clinical heterogeneity, which is an inherent problem in data synthesis. The emergency settings in the rescuing from cardiac arrest bring particular difficulties of access to the expected homogeneity in clinical studies. The reviewed studies included patients with varied baseline characteristics (such as the settings of collapse, basic diagnosis, bystander CPR, CPR protocols performed by professional rescuers, and different regimens of antiarrhythmics and postresuscitation care, such as hypothermia). Although no significant difference was found in baseline characters between the study groups, all these variables lead to clinical heterogeneity. Significant statistical heterogeneity was found according to the χ*2* test and *I*^*2*^ statistic in subgroups. These encouraged us to use a random-effect model.

So according to these issues, the pooled results of the data from current evidence still need to be interpreted with caution. We assessed the data according to subgroups of OHCA and IHCA, but conversely, the sample size is rather weakened.

Our review provides some implications. To our knowledge, lack of evidence focused on medications during CPR can be found. No antiarrhythmia agents have been demonstrated to improve the survival of cardiac-arrest patients. According to studies of high quality, only amiodarone seems to be beneficial in the improvement of initial resuscitation, although the strength of existing evidence is not robust enough, according to the sample size. Our review supports the summarization of the effects of antiarrhythmics and the recommendation of medication strategies in the AHA 2010 guidelines [5,6], and we suggest the use of amiodarone in patients with refractory VF/pulseless VT. A previous review provided a similar implication. Compared with the review, we performed a more up-to-date literature search; we also used an optimized tool to assess the quality of evidence, and we synthesized data by meta-analysis.

According to the review, a major issue of research in resuscitation drugs is the lack of clinical data. It is important to develop randomized prospective studies with large sample sizes to assess the effects of antiarrhythmia agents during CPR. According to our review, nonrandomization and nonblinding design seem to bring a high risk of selection bias and performing bias, which might affect the study results. Larabee *et al*. [28] suggested that developing prospective regional/national systems for analysis of cardiac arrest outcomes would be help to capture an appropriate sample size to examine the medications and intended outcomes.

We suggest that it is important for the articles to report details about the randomization or blinding methods (such as allocation concealment) in resuscitation studies, because in the research into resuscitation, the details of randomization and blinding are important in the assessment of the quality of evidence. Additionally, no new drugs seem to be valuable in resuscitation currently. Studies have not applied new antiarrhythmics to resuscitation, such as other potassium-channel blockers, but it is possible that these agents (such as ibutilide, dofetilide) may have the potential to be used during CPR, according to the pharmacologic mechanisms. Further studies focusing on these agents might also be expected.

## Conclusions

In summary, no current drugs have been shown to improve the survival in cardiac-arrest patients. Only amiodarone may improve initial resuscitation, and no new drugs can be recommended currently. A major issue in resuscitation research is that relevant studies focused on resuscitation drugs are very limited, especially high-quality studies.

## Key messages

● An up-to-date systemic review and meta-analysis have found that according to present literature, only limited data about the use of antiarrhythmic drugs for cardiac arrest can be found, and the quality of evidence is rather low. No antiarrhythmic drugs have been shown to improve the survival of cardiac-arrest patients, and only amiodarone is suggested to be used for improving initial resuscitation by our review.

● More clinical studies focused on resuscitation drugs are expected and warranted, especially studies with good quality and focused on new types of antiarrhythmic drugs.

## Abbreviations

ACLS: Advanced cardiac life support; AHA: American Heart Association; CI: Confidence interval; CPC: Cerebral performance category; EMS: Emergency medical service; IHCA: In-hospital cardiac arrest; ILCOR: International Liaison Committee on Resuscitation; OHCA: Out-of-hospital cardiac arrest; RCT: Randomized controlled trial; ROSC: Return of spontaneous circulation; RR: Risk ratio; SCA: Sudden cardiac arrest; VF: Ventricular fibrillation; VT: Ventricular tachycardia.

## Competing interests

We received funding support from National Natural Science Foundation of China (NSFC) 81071539. The authors declare that they have no competing interests.

## Authors’ contributions

YH and QH were responsible for study concept and design. YH acquired the data. YH, MY, LZ, and QH analyzed and interpreted the data. YH and QH performed statistical analysis. YH drafted the manuscript. QH was responsible for the revision of the manuscript for important intellectual content. QH supervised the study. YH, MY, LZ, and QH read and approved the manuscript for submission.

## Authors’ information

Yu Huang and Qing He are co-first authors.
